# Molecular and serological investigation of Hepatitis E virus in pigs slaughtered in Northwestern Italy

**DOI:** 10.1186/s12917-023-03578-4

**Published:** 2023-01-25

**Authors:** Emanuele Carella, Francesca Oberto, Angelo Romano, Simone Peletto, Nicoletta Vitale, Annalisa Costa, Claudio Caruso, Laura Chiavacci, Pier Luigi Acutis, Ledi Pite, Loretta Masoero

**Affiliations:** 1 Istituto Zooprofilattico Sperimentale del Piemonte, Liguria E Valle d’ Aosta, Via Bologna 148, 10154 Turin, Italy; 2 Azienda Sanitaria Locale CN2, Via Gerolamo Vida 10, 12051 Alba (CN), Italy; 3 Azienda Sanitaria Locale CN1, Via Pier Carlo Boggio 12, 12100 Cuneo, Italy

**Keywords:** Hepatitis E virus, ELISA, Real-time RT-PCR, Abattoir, Swine, Muscles

## Abstract

**Background:**

Hepatitis E Virus (HEV) is recently considered an emerging public health concern. HEV genotypes 1 and 2 are widely distributed and pathogenic only for humans. In contrast, HEV, genotypes 3 and 4 are observed in swine, deer, wild boars and rabbits and can also be transmitted to humans. The presence of HEV in the liver, muscle, faeces, blood, and bile was detected by real-time RT-PCR in 156 pigs belonging to twenty different farms, ranging from 1 to 8 months of age. The phylogenetic analysis was performed on the viral strain present in the positive biological matrix, with the lowest Ct. HEV-IgG and HEV-IgM in the sera were analysed by two different ELISA kits.

**Results:**

Twenty-one pigs, i.e., 13.46% of them (21/156, 95% CI: 8.53%-19.84%), tested positive for HEV in at least one biological matrix by real-time RT-PCR, while phylogenetic analysis revealed the presence of HEV subtypes 3f and 3c. Pig serums analysed by ELISA showed an overall prevalence of 26.92% (42/156, 95% CI: 20.14%-34.60%) for HEV-IgG, whereas the 28.95% (33/114, 95% CI: 20.84%-38.19%) of them tested negative resulted positive for the HEV-IgM.

**Conclusions:**

The faeces are the biological matrix with the highest probability of detecting HEV. The best concordance value (Kappa Kohen index) and the highest positive correlation (Phi index) were observed for the correlation between bile and liver, even when the number of positive liver samples was lower than the positive bile samples. This finding may suggest that a higher probability of HEV occurs in the bile, when the virus is present in the liver, during the stages of infection. Finally, the presence of HEV in muscle was observed in 11 pigs, usually used for the preparation of some dishes, typical of the Italian tradition, based on raw or undercooked meat. Therefore, their consumption is a possible source of infection for final consumer.

## Introduction

Hepatitis E Virus (HEV) is a”quasi-enveloped” positive sense single-stranded RNA virus and is classified in eight different genotypes included in the family *Hepeviridae*, genus *Orthohepevirus* [1]. HEV is responsible for severe and rarely fatal diseases in humans. The mortality rate usually ranges from 1 to 4% [2]. In pregnant women, HEV infection has been associated with up to 30% mortality in the third trimester [3]. HEV genotypes 1 and 2 (g1 and g2) are widely distributed and pathogenic only for humans. HEV genotypes 3 and 4 (g3 and g4) are observed in swine, deer, wild boars, and rabbits and can also be transmitted to humans. HEV genotypes 5 and 6 (g5 and g6) are detected only in swine [4–6]. In the Suidae family asymptomatic infection generally occurs and the viraemia is of short duration [7]. Pigs and wild boars are usually considered “reservoirs”. Therefore, these animal species are fundamental for a better understanding of the spread of HEV, even though new animal hosts and viral strains are recently reported [8, 9].

The main zoonotic transmission route for HEV is the faecal‐oral route through water contaminated with faeces [6]. Zoonotic transmission usually occurs in pigs of intensive farms, and strains are usually genetically related to those isolated in humans [4]. However, HEV also spreads through blood transfusion, organ donation and food-borne route, linked to the consumption of raw pork foodstuffs [4]. In fact, raw or undercooked pork meat or pork liver sausages are the most frequently reported food products associated with sporadic cases or outbreaks of HEV. In addition, infected animals are carriers of the virus that can be shed at a high level in faeces and bile. These biological matrices could represent a possible vehicle for food cross-contamination during slaughter, evisceration and food processing [4]. Therefore, infected suids represent a public health concern, as sources of exposure for human consumers also after low temperature thermal treatment [4, 10]. In Italy, after five years of surveillance in humans (2012–2016), 3.34% of cases of acute hepatitis were linked to HEV strains, and the highest seropositivity rate in blood donors was detected in Central Italy (22.8%) [11]. Despite a low number of clinical human cases of Hepatitis E in industrialised Countries, a relatively high seroprevalence has been detected, suggesting that the HEV infection in humans could be underestimated [12, 13]. Previous studies have already investigated the presence of HEV using PCR and ELISA in various abattoirs, suggesting that the level of prevalence is highly variable according to the geographical areas and the age of the animals included in the sampling [7, 14]. Molecular analysis of organs and tissues involved in the infection and the excretion of the virus represents the best diagnostic tool for epidemiological investigations [7, 12].

In Northwestern Italy, the swine industry is an important business with 2880 farms and an overall of 1.164095 million animals, mostly located in the Piedmont region [14]. Besides the main production of adult pigs, slaughtered with an average weight of 160 kg, some local abattoirs focus their activity on the younger swine population. In particular, the pigs, slaughtered with a maximum body weight of 110 kg, are important for some typical traditional Italian dishes.

The aim of this study was to investigate the serological and molecular prevalence of HEV in pigs slaughtered at three different abattoirs in the Piedmont region. Five biological matrices such as blood, muscle, liver, bile, and faeces were collected from 156 pigs and analysed to detect the presence of HEV by real-time RT-PCR. Phylogenetic analysis of the positive samples was performed to characterise and identify HEV subtypes present in the study area. A serological investigation for HEV-IgM and HEV-IgG was also performed to analyse the presence of maternal or active immune response against HEV.

## Material and methods

### Abattoirs selection and cross-sectional study

Piedmont is located in Northwestern Italy and is the third Italian region important by the number of swine farms (11% of Italian production) [14]. Italian pig production is different from the other European countries due to its long production cycle. Weaners between 8 and 25 kg, growers between 25 and 70 kg and finishers, between 70 and 110 kg are slaughtered for fresh meat consumption (from 1 to 8 months). The adult pigs, that produce Protected Designation of Origin ham (Prosciutto di Parma, Prosciutto di Cuneo), are usually slaughtered at nine months with an average weight of 160 kg.

The present investigation was designed as a prevalence cross-sectional study. Three different abattoirs located in the Piedmont region, which slaughter only pigs, with a body weight ranging from 13 to 110 kg, were selected for the study. 26 animals were sampled from the first abattoir, 63 from the second abattoir and 67 from the third abattoir. Therefore, a total of 156 pigs, belonging to 20 different farms, ranging from 1 to 8 months of age, were analysed.

The sample size was determined considering a 95% confidence interval, an expected prevalence of 50%, and an error of 8%. No correction for the finite population was used as the population target consist of 248,076 animals, within the study area [15]. The number of animals to analyse, for each abattoir, was determined on the different numbers of weekly-slaughtered pigs.

### Sample collection

The blood, the liver, the bile, and the faeces were collected from the target population of the three abattoirs. The abattoirs workers were located at different points along the slaughter chain, to collect the five biological matrices (blood, liver, bile, cecum and muscle) analysed for each sampled animal. Blood was collected during the animal's bleeding using special test tubes (with and without anticoagulant) previously marked with the animal's identification number and then stored at 4 °C. A lobe of the liver and the entire gallbladder, together with the cecum, were collected during the evisceration phase. From the cecum, the faeces were subsequently sampled. Finally, a portion of neck muscles was removed from the carcass in the phase before refrigeration. All the biological matrices collected were then placed in a sterile disposable plastic bag. The bile of two animals was not available and the blood samples were collected in EDTA tubes and stored at 4 °C. The muscle samples were collected to investigate the co-presence of HEV in animals that previously tested positive in the other biological matrices. The traceability of the biological samples was ensured during the entire slaughter chain and all the biological samples were stored at -80 °C after the collection in the abattoirs.

### RNA extraction and real-time RT-PCR conditions

The 10% weight/volume suspensions were prepared in PBS for liver, bile and faeces, whereas the blood samples were analysed undiluted. Then 350 µl of the suspensions were used for the RNA extraction thanks to the TRI Reagent® (Sigma-Aldrich, Darmstadt, Germany) and the QIAamp® Viral RNA Mini Kit (Qiagen, Hilden, Germany) following the manufacturer’s protocol. Instead, the RNA extraction from the muscle samples was performed according to Chelli et al. [16]*.*

The detection of HEV in the liver, faeces, bile, and blood was performed by real-time RT-PCR with the CFX96 Touch™ Real-Time PCR Detection System (Biorad, Hercules, USA). Detection employed the TaqMan approach, targeting the ORF3 genomic region. A real-time RT-PCR protocol was adopted from Jothikumar et al. [17] with primers and TaqMan probe manufactured by Thermo Fisher Scientific (Waltham, USA). The amplification of HEV extracted from the muscles was performed according to Chelli et al. [16]*.* In detail, the reaction volume of 25 μl consisted of 5 μl of template, 5 µl of RNA Ultrasense™ reaction mix (Invitrogen, Waltham, USA), 500 nM of primer Forward JVHEVF (5′-GGTGGTTTCTGGGGTGAC-3′), 900 nM of primer Reverse JVHEVR (5′-AGGGGTTGGTTGGATGAA -3′), 250 nM of Taqman Probe JVHEVP-MGB (5′-FAM-TGATTCTCAGCCCTTCGC-MGB-3′), 1,25 µl of RNA Ultrasense enzyme mix (Invitrogen, Waltham, USA) and nuclease-free water up to the final volume. The assay was carried out with the CFX96 Touch™ Real-Time PCR Detection System (Biorad, Hercules, USA), using the following PCR cycling conditions: 1 cycle of reverse transcription at 50 °C for 1 h, 1 cycle of PCR initial activation step at 95 °C for 5 min followed by 45 cycles of 95 °C for 15 s, 60 °C for 1 min and 65 °C for 1 min.

### Phylogenetic analysis

When a pig is HEV positive for more than one biological matrix, only that with the lowest Ct, determined through real-time RT-PCR, is used for reverse transcription to cDNA and phylogenetic analysis. Reverse transcription was carried out using One Script cDNA Synthesis kit (ABM, Richmond, Canada). The reaction was performed in a total volume of 20 μl, containing 4 μl RT buffer (5 ×), 1 μl dNTPs (10 µM), 1 μl Random primers, 1 μl One Script RTase (200 U/μl), 0.5 μl Rnase Off Ribon. Inhibitor (40 U/μl), and 7 μl RNA. The thermal profile was performed as follows: 10 min at 25 °C, 50 min at 42 °C, and 5 min at 85 °C.

The phylogenetic analysis was performed comparing the 5’ region of the ORF2 gene, targeting a variable region that provides a phylogenetic signal comparable to full genome analysis [18]. A Nested PCR assay was performed using an external primer set (3156N forward, 3157 reverse) for a first amplification round (710 bp) and an internal primer set (3158N forward, 3159 reverse) for a second ones (348 bp) [19]. The assay was carried out in a final volume of 50 μl with 1X Maxima Hot Start Taq buffer, 1.5 mM MgCl_2_, 0.2 mM of each dNTP, 0.2 μM of each primer, and 1U of Maxima Hot Start Taq DNA Polymerase (Thermo Fisher Scientific, Waltham, USA). The following thermal conditions were applied: activation of Taq polymerase at 95 °C for 4 min, followed by 40 cycles of denaturation at 95 °C for 1 min; annealing at 60 °C for 1 min; extension at 72 °C for 2 min; and final elongation at 72 °C for 7 min. Amplification products were checked by electrophoresis on 2% agarose gel, purified with the Extract me DNA Clean-up (Blirt, Gdańsk, Poland) and sequenced using a BrilliantDye Terminator v3.1 cycle sequencing kit (NimaGen, Nimega, Netherlands). The amplicons were purified with the DyeEx 2.0 Spin Kit (Qiagen, Hilden, Germany) and run on a 3130xl Genetic Analyzer (Life Technologies, Carlsbad, USA). A dataset of partial sequences of 135 bp of the 5’ ORF2 region from the analyzed samples and 151 GeneBank accessions have been aligned. The phylogenetic analysis was performed using MEGA7 software with the Neighbour-Joining method and the Kimura-2 model. Statistical robustness and reliability of the branching order were confirmed by bootstrap analysis using 1,000 reiterations [20].

### Serological analysis

The blood samples were also collected to extract the serum through centrifugation at 3500 g for 10 min and then stored at -20 °C until the serological analysis. A commercial ELISA kit, specific for the detection of HEV-IgG in animal serum samples, was employed according to the manufacturer’s instructions (ID screen®, Grabels, France). The plate absorbance was read at a wavelength of 450 nm and the Net OD values of the samples were calculated and expressed as percentages of reactivity (pOD) of the plate positive control. The samples with percentages of reactivity (pOD) higher than 70% were classified as positive, samples with pOD between 60 and 70% as doubtful and samples with pOD lower than 60% were considered as negative. Serums that resulted as doubtful were tested in a second test session.

No commercial ELISA kit was available on the market exclusively to detect HEV-IgM in swine serums, while the tests were being carried out. Therefore, the HEV total antibody ELISA kit (Wantai Biopharm, Beijing, China), which can detect both HEV-IgG and HEV-IgM in animal sera, was used for the 114 serums tested negative for HEV-IgG, according to the manufacturer instructions. The plate was read at the wavelength of 450 nm and the absorbance of the blank well was subtracted from the absorbance values of specimens and controls. The cut-off calculation was performed by adding the value 0.12 to the average absorbance for three negative plate controls. Samples with a ratio ≥ 1 (sample OD/cut-off) were considered positive, samples with a ratio between 0.9 and 1.1 were considered doubtful and samples with a ratio lower than 0.9 were classified negative. Serums that resulted as doubtful were tested in a second test session.

### Statistical analysis

The proportion of positives was calculated for each matrix. The binomial distribution was used to calculate the exact confidence limit of each proportion. The correlation index and Cohen’s Kappa index were used to assess the agreement between the biological matrices [21]. The Kappa index indicates the proportion of agreement, excluding that expected by chance, for categorical variables. Kappa values near 1 indicate perfect agreement, while a Kappa value of 0 indicates that all the agreement is due to chance. According to Landis et al*.* [22] the value of Kappa index between 0.60 and 0.8 was considered good agreement.

The Phi index indicated correlations between matrices, i.e., a measure of association for two binary variables. Values of Phi range from − 1 to + 1, where 1 indicates perfect agreement, -1 perfect disagreement, and 0 indicates no relationship [23]. All statistical analyses were performed by SAS System v 9.4.

To evaluate the agreement between all five matrices, Fleiss' Kappa, as an index of interrater agreement between matrices was calculated [24], using package irr (version 0.84. 1) [25].

## Results

HEV was detected in 21 pigs with an overall prevalence of 13.46% (21/156, 95% CI: 8.53%-19.84%) in at least one biological matrix, by real-time RT-PCR. Faecal and bile samples resulted positive for HEV in 90.48% (19/21, 95% CI: 69.62%-98.82%) and in 61.90% (13/21, 95% CI: 38.44%-81.89%;) of the positive pigs, respectively. The prevalence of HEV in the liver and the blood samples was 38.09% (8/21, 95% CI: 18.11%-61.56%) and 14.28% (3/21, 95% CI: 3.05%-36.34%) respectively. The prevalence of HEV in the neck muscles was 52.38% (11/21, 95% CI: 29.78%-74.27%) (Table 1). All the pigs that tested positive for HEV were between 1 and 3 months of age and had body weights ranging from 12 to 45 kg.

**Table 1:** Ct values of the biological matrices tested positive for HEV (blood, liver, bile, faeces, and muscle), serological outcomes and titers (HEV IgG and IgM-IgG) of the twenty-one positive pigs and genotype of the viral strains detected in the matrix with the lowest Ct value

ID Sample	pig farm	abattoirs	weight (kg)	Age (days)	Blood	Liver	Bile	Faeces	Muscle	IgG	IgG-IgM	genotype
1	7	3°	45	75	-	31	28	32	35	+ (186%)	•	3f*
2	7	3°	45	75	35	28	27	25	31	- (8%)	+ (1.8)	3f*
3	7	3°	45	75	-	31	28	35	33	+ (178%)	•	3f*
4	7	3°	45	75	-	30	29	31		+ (247%)	•	3f*
5	7	3°	45	75	-	-		29		- (52%)	+ (8.3)	3f*
6	7	3°	45	75	-	31	30	36	32	- (6%)	+ (8.8)	3f*
7	9	2°	25	55	-	-		31		+ (202%)	•	•
8	9	2°	25	55	-	-		31		+ (257%)	•	3f
9	8	3°	12,5	45	-	-	36			+ (128%)	•	•
10	10	3°	15	37	38	27	20	24	32	+ (110%)	•	3f
11	10	3°	15	37	-	-	33			- (10%)	- (0.2)	3f*
12	11	3°	13	40	-	-		35	29	- (20%)	+ (4.4)	•
13	11	3°	13	40	-	-		32		- (14%)	+ (3.7)	3f
14	15	1°	30	80	-	32	30	27		+ (192%)	•	3c
15	17	1°	35	70	35	33	33	24		+ (218%)	•	3f*
16	17	1°	35	70	-	-		32	31	+ (205%)	•	3c*
17	19	2°	19	50	-	-	32	33	35	- (1%)	- (0.9)	•
18	19	2°	19	50	-	-	31	34	29	- (2%)	- (0.1)	3f*
19	19	2°	19	50	-	-	33	34	35	- (1%)	- (0.5)	•
20	17	1°	35	90	-	-	-	33	31	+ (208%)	•	•
21	17	1°	35	90	-	-	-	33		+ (201%)	•	3c*

+ —positive outcome; - —negative outcome; *• *—not tested sample; * closely related strains, shown in Fig. 2

**Fig. 1:**
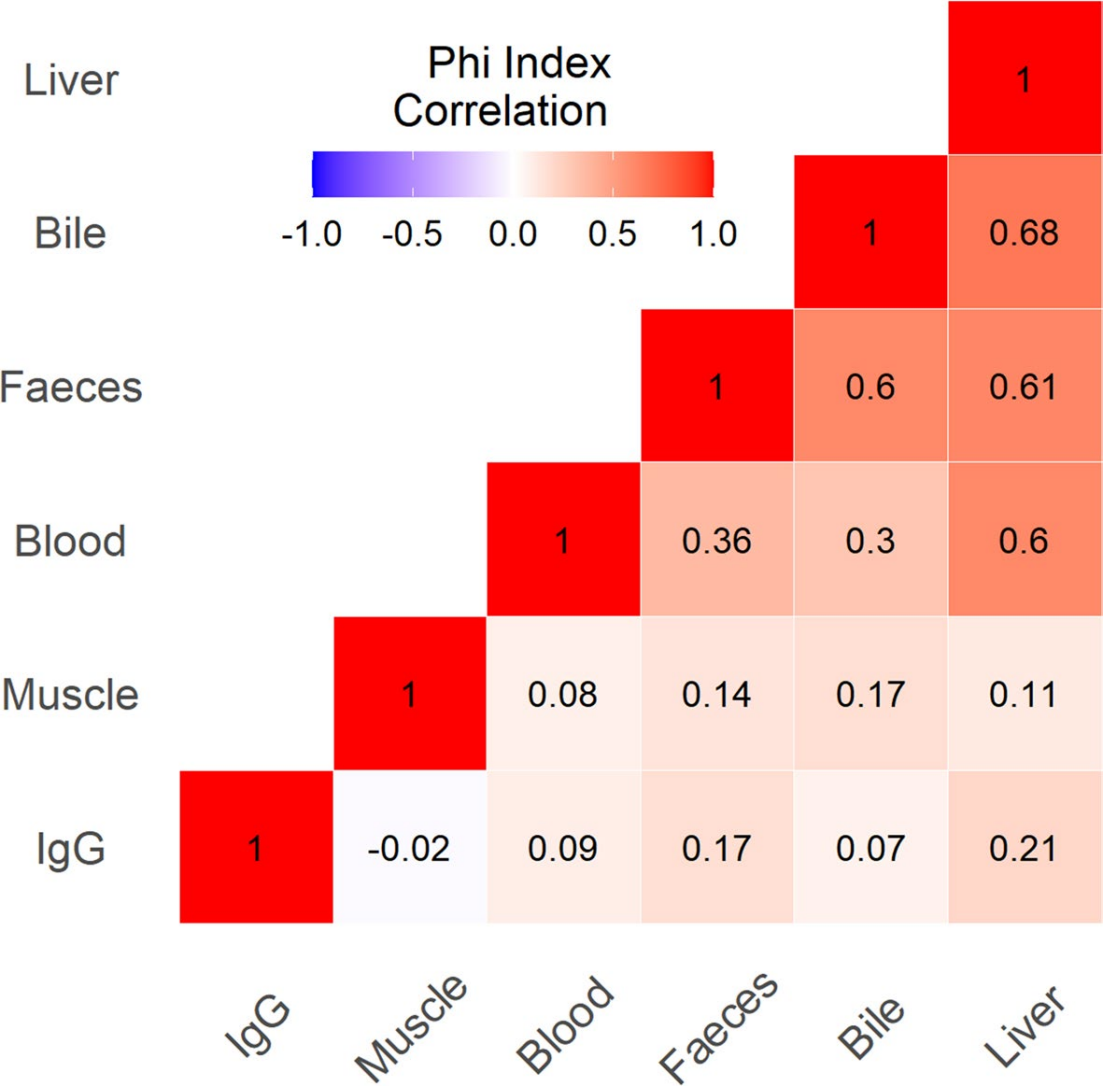
Heat map of the Phi correlation index ranging from 1 to -1. Red colour highlights a strong positive association and a high level of agreement (darker reds evidence a stronger association) concerning blue colour that shows a negative association and disagreement. White colour (Phi = 0) evidence of no association and no agreement

The Kappa index for each couple of matrices was shown in Table 2. The best concordance values were detected, respectively, for the correlation between bile and faeces (0.61) and between bile and liver (0.66). Instead, a mild concordance was observed between liver and blood (0.54). In addition, the Phi index, i.e., a measure of association for two binary variables, was determined for each pair of matrices investigated (Fig. 1). The highest positive correlation between bile/liver was observed (Phi index 0.68) while no correlation between IgG and muscle was found (Phi index -0.02). The presence of HEV-IgG was also not positively associated with faeces blood, liver and bile, while a mild concordance was observed between liver and faeces.

**Table 2:** Determination of the agreement between two biological matrices expressed as Kappa Cohen index

Biological matrices	Kappa index	CI95% KAPPA		agreement
Bile-faeces	0.61	0.41	0.81	good
Bile-liver	0.66	0.42	0.89	good
Bile-muscle	0.13	0.02	0.23	poor
Bile-blood	0.24	-0.04	0.52	low
Faeces-muscle	0.16	0.05	0.27	poor
Faeces-blood	0.26	0.02	0.50	low
Liver-muscle	0.08	-0.01	0.17	No agreement
Liver-blood	0.54	0.18	0.90	mild
Liver-faeces	0.58	0.36	0.80	mild
Blood-muscle	0.04	-0.02	0.09	No agreement
Bile-IgG	0.12	-0.03	0.27	poor
Faeces-IgG	0.23	0.07	0.40	low
Liver-IgG	0.17	0.03	0.31	poor
Blood-IgG	0.05	-0.04	0.15	No agreement

Above 0.60 the agreement is considered good (i.e. bile/faeces or bile/liver), between 0.40 and 0.60 is considered mild (i.e. liver/blood), between 0.20- 0.39 is considered low (i.e. bile/blood, faeces/blood), between 0.19–10 is considered poor agreement and < 0.1 there is no agreement

The total agreement between all five matrices resulted low as the Fleiss’ Kappa was 0.212 (95% CI: 0.15- 0.27).

**Fig. 2:**
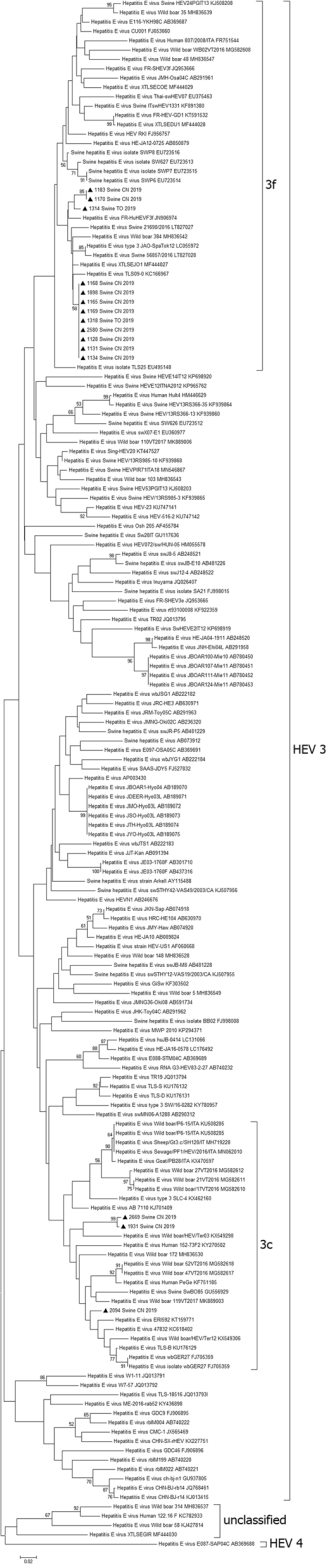
Phylogenetic tree based on the 5’ end of the ORF2 sequence of HEV and constructed using the Neighbour-Joining method and the Kimura-2 model. HEV isolates are indicated by black triangles and the HEV subtypes for genotype 3 are identified outside the brackets. Bootstrap analysis values (percentages) are shown. The bar at the base of the tree shows the scale for nucleotide substitution per site

Sequencing and phylogenetic analysis were successful in 15 out of 21 positive animals and the phylogenetic tree is shown in Fig. 2. The other positive samples exhibited genetic sequences of low quality, even after repeated attempts, and so were not taken into consideration for phylogeny. HEV subtype 3f was the most prevalent genotype detected (12 out of 15 positive pigs) while HEV subtype 3c was observed in the other three animals. The subtypes 3f and 3c were also observed in two different animals belonging to the same farm (Table 1). Overall similarity among the 15 HEV strains, for which phylogenetic analysis was possible, ranged from 81.5% to 100%.

**Fig. 3:**
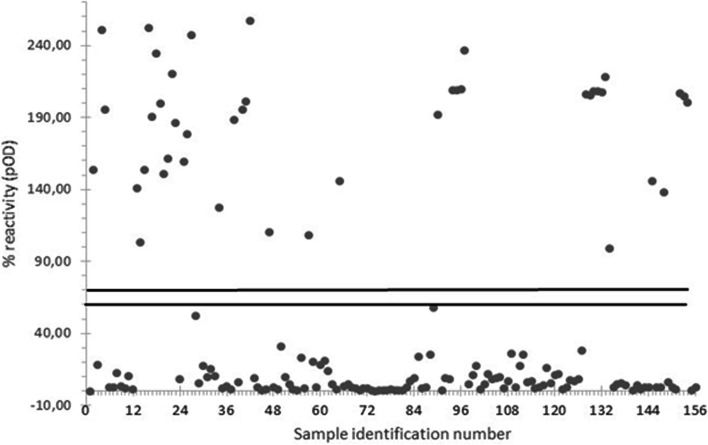
ELISA results of HEV-IgG performed in serum samples collected from 156 slaughtered pigs (grey points). Samples absorbance was expressed as the percentage of absorbance(pOD) in comparison with the absorbance of the positive control of the plate. Samples with a pOD higher than 70% are considered positive, while samples with a pOD lower than 60% are detected as negative. The bold black lines delimit the doubtful range of the test

**Fig. 4:**
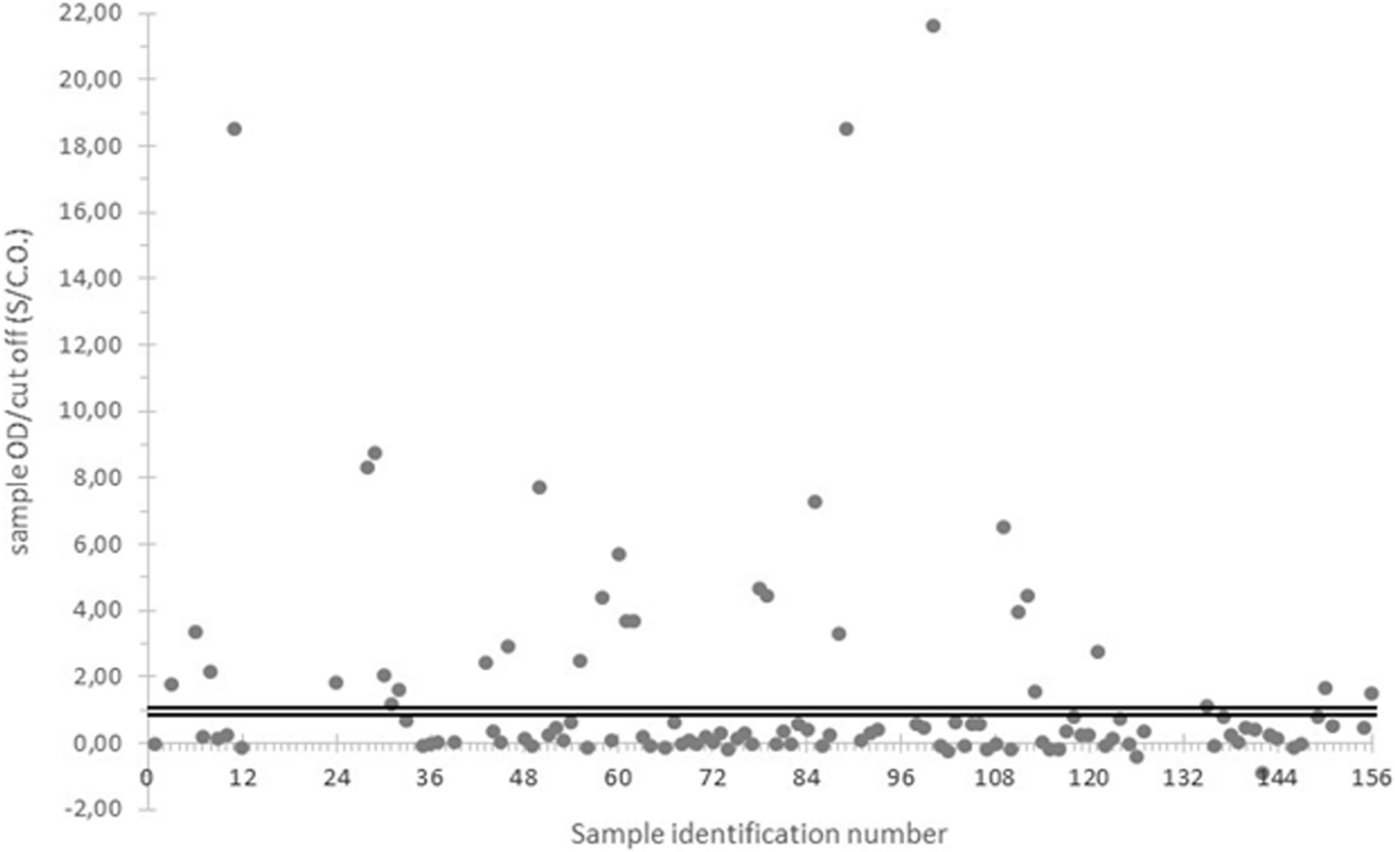
ELISA results of HEV-IgM performed in serum samples collected from the 114 animals tested negative for HEV-IgG (grey points). The Cut-off value (C.O.) was calculated by adding 0,12 to the mean value of the negative control in the plate. Among the serums tested, those with the rate S/C.O. (Sample OD/Cut Off value) lower than 0.9 were classified as negative and those with the rate S/C.O. higher than 1.1 as positive. The doubtful range is delimited by the bold black lines

The detection of HEV-IgG, by ELISA, revealed that 26.92% of the investigated pigs (42/156, 95% CI: 20.14%-34.60%) have detectable levels of HEV antibodies (Fig. 3). The presence of HEV-IgM was also investigated in the 114 animals that tested negative for HEV-IgG. A percentage of 28.95% (33/114, 95% CI: 20.84%-38.19%) IgM positive serums was detected (Fig. 4).

Regarding 21 HEV positive animals, 12 resulted positive for HEV- IgG, 5 tested positive for HEV-IgM and the remaining 4 pigs were negative both for HEV-IgG and HEV-IgM. Among the three viremic animals, which resulted positive for HEV in blood, two specimens presented still detectable levels of IgG and the other was negative for HEV-IgG and positive for HEV-IgM (Table 1).

## Discussion

Industrialized countries were traditionally considered HEV non-endemic areas with only sporadic cases. In Italy, several studies have been performed to determine the prevalence, the diffusion and risk of zoonotic HEV infection in swine [7, 11, 16, 26, 27]. In the Piedmont region, the swine industry currently includes two different populations with different weights and ages. A previous study investigated the presence of viral RNA in faecal samples and detectable levels of HEV-IgG in forty-two adult swine farms located in the Piedmont region. The seropositivity rate was 50.23%, while HEV in the faeces was 10.9% of the tested swine [14]. The slaughter of piglets has been reported as a possible source of infection both for slaughterhouse workers and final consumers of raw or undercooked pork meat [28]. The liver and muscles are often used for the preparation of typical Italian dishes, based on raw meat such as fresh sausages, even though being involved as a source of infections in human consumers [7, 29].

This study was carried out in an “intensive pig farming area” and focused on a swine population, potentially involved in the transmission of HEV. In Italy, the meat of the weaners, growers and finishers is usually sold in the supermarket, therefore, their sampling could be useful to better understand the dynamics of the HEV-infections, through foodborne route. In Piedmont, no data are currently available about the presence of HEV in several biological matrices and HEV-IgM and HEV-IgG in sera of pigs with a maximum age of 8 months.

The faecal samples had the highest probability of detecting the HEV virus (19/156 animals), confirming the long faecal shedding of the virus in infected pigs as already reported in previous studies [28, 30]. The best concordance value (the Kappa Kohen Index 0.66) and the highest positive correlation (the Phi index 0.68) were observed between bile and liver, even if the number of positive liver samples (8/156) is lower than the positive bile samples (13/156). This finding is consistent with other studies where the bile was one of the most frequently positive biological matrices in swine [31, 32]. Moreover, HEV was observed for longer periods and at a higher frequency compared to the liver [29]. These findings may suggest that a higher probability of HEV occurs in the bile when the virus is present in the liver during the stages of infection. The bile was the only infected matrix in two pigs, suggesting that the accidental rupture of the gallbladder may affect the transmission of the HEV in other biological matrices, during the slaughtering procedure. Therefore, faeces and bile are likely the biological matrices more suitable to detect the presence of HEV in swine population.

The phylogenetic analysis shows that all identified HEV isolates belong to genotype 3 with a zoonotic potential that is linked to the ingestion of raw meat from infected animals [27]. HEV isolates from the pigs in the Piedmont region are classified into subtypes 3c, 3e, and 3f [14]. Our results are consistent with those previously reported since 12 and three isolates were classified into subtypes 3f and 3c, respectively. The subtype 3f, i.e., one of the predominant autochthonous phylogenetic groups in Europe [17] has already been reported in Northwestern Italy [14]. This subtype results in a higher risk of hospitalisation compared to other HEV genotypes 3 [33]. However, also the subtype 3c has frequently been detected in European countries [34]. These two subtypes were also observed in a wild boar population located in Northwestern Italy [35, 36]. They could spread the virus to other animal species since the HEV-3 genotypes are characterized by cross- species transmission [37].

Regarding the serological analysis, the humoral response to the virus, due to a previous HEV infection or the residual maternal immunity can explain the presence of detectable levels of HEV-IgG antibodies in 26.92% (42/156) of the investigated pigs. Therefore, both the presence of HEV-IgM and the absence of HEV IgG, in thirty-three animals at the same time, could indicate a recent viral infection and an early immune response. This finding is in concordance with previous studies where a clear correlation between the levels of anti-HEV antibodies in the sows and in the piglets was observed [28, 38].

Among 21 pigs tested positive for HEV by real-time RT-PCR, the 80.95% (17/21) of the specimens showed a detectable level of HEV antibodies in serum samples (HEV–IgG or HEV-IgM). 12 out of 21 infected animals tested positive for HEV IgG, five animals scored positive for HEV-IgM and four pigs were negative for both immunoglobulins. Nevertheless, it was not possible to determine a close association (Phi index) between the presence of IgG in the serum and HEV in the different biological matrices. Viremia is detectable only during a short period after infection [38]; therefore, all the pigs probably developed viremia, but because of the timing of sampling, only 1.92% (3/156) of the specimens were observed. This finding is inconsistent with recent study where the percentage of viremic pigs was higher compared to our results [16, 39]. However, a not negligible risk of accidental infection for slaughterhouses workers could also occur through the blood of viremic animals. [40, 41].

The detection of HEV in muscles of naturally infected pig has been reported in some reports, but only in swine older than those analysed in our study [42–44]. Regarding the piglets, HEV was observed in different biological matrices, but not in the muscles [28]. In this study, the presence of HEV in the muscles of weaners and growers (from 1 to 3 months) was instead observed in 11 out of 21 positive samples. The European Food Safety Authority (EFSA) has pointed out, as a priority for future research projects, the optimisation and standardization of HEV isolation in cell cultures, to evaluate the onset of clinical cases in humans and the risk of exposure [4]. Therefore, further investigation to confirm the presence of the virus in the muscle should be performed, such as the HEV isolation in cell cultures, even if it requires long and expensive laboratory procedures.

## Conclusion

In conclusion, this study has provided insight into the status of HEV infection in pig slaughtered in an intensive pig farming area, located in north-western Italy. Regarding the biological matrices investigated, the faeces are the biological matrix with the highest probability of detecting HEV, confirming the long faecal shedding of the virus in infected pigs. The bile was the unique infected matrix in two pigs and the number of positive liver samples was lower than the positive bile samples. This finding suggests a higher probability of the HEV occurs in the bile, when the virus is present in the liver, during the stages of infection.

The presence of HEV in muscles was also observed in weaners and growers. The infected muscle has only been reported only in swine older than those analysed in our study. Further investigation to confirm the presence of the virus in the muscle should be performed, such as the HEV isolation in cell cultures.

Serological data confirmed an active circulation of HEV in the investigated farms. The humoral response to the virus, due to a previous HEV infection or the residual maternal immunity can explain the presence of detectable levels of HEV-IgG antibodies in 26.92% of the investigated pigs. Instead, the presence of HEV IgM and the absence of HEV IgG, in 33 animals at the same time, could suggest a recent viral infection and an early immune response.

## Data Availability

All data are available from the corresponding author on reasonable request. The datasets generated and analysed during the current study are available in the GenBank repository under accession numbers from OQ067485 to OQ067499, at the following links: from https://www.ncbi.nlm.nih.gov/nuccore/OQ067485.1/ to https://www.ncbi.nlm.nih.gov/nuccore/OQ067499.1/.

## Abbreviations

HEV: Hepatitis E virus
ELISA: Enzyme Linked ImmunoSorbent Assay
EDTA: EthylenDiaminoTetracetyc Acid
RT-PCR: Reverse transcript- polymerase chain reaction
